# Reduced SHARPIN and LUBAC Formation May Contribute to CCl_4_- or Acetaminophen-Induced Liver Cirrhosis in Mice

**DOI:** 10.3390/ijms18020326

**Published:** 2017-02-04

**Authors:** Takeshi Yamamotoya, Yusuke Nakatsu, Yasuka Matsunaga, Toshiaki Fukushima, Hiroki Yamazaki, Sunao Kaneko, Midori Fujishiro, Takako Kikuchi, Akifumi Kushiyama, Fuminori Tokunaga, Tomoichiro Asano, Hideyuki Sakoda

**Affiliations:** 1 Department of Medical Science, Graduate School of Medicine, University of Hiroshima, 1-2-3 Kasumi, Minami-ku, Hiroshima City, Hiroshima 734-8551, Japan; ymmty@hiroshima-u.ac.jp (T.Y.); nakatsu@hiroshima-u.ac.jp (Y.N.); ymatsunaga@hiroshima-u.ac.jp (Y.M.); 2 CellBiology Unit, Institute of Innovative Research, Tokyo Institute of Technology, 4259-B16 Nagatsuta, Midori, Yokohama 226-8501, Japan; tofu@bio.titech.ac.jp; 3 Division of Diabetes and Metabolism, The Institute for Adult Diseases, Asahi Life Foundation, Chuo-ku, Tokyo 103-0002, Japan; hiyamazaki-tky@umin.ac.jp (H.Y.); kikuchi-tk@umin.ac.jp (T.K.); kusiyaa-tky@umin.ac.jp (A.K.); 4 Department of Internal Medicine, Graduate School of Medicine, University of Tokyo, 7-3-1 Hongo, Bunkyo-ku, Tokyo 113-8655, Japan; kanekos-tky@umin.ac.jp; 5 Division of Diabetes and Metabolic Diseases, Nihon University School of Medicine, Itabashi, Tokyo 173-8610, Japan; midori-tky@umin.ac.jp; 6 Laboratory of Pathobiochemistry, Graduate School of Medicine, Osaka City University, 1-4-3 Asahi-machi, Abeno-ku, Osaka City, Osaka 545-8585, Japan; ftokunaga@med.osaka-cu.ac.jp; 7 Division of Neurology, Respirology, Endocrinology and Metabolism, Department of Internal Medicine, Faculty of Medicine, University of Miyazaki, 5200 Kihara, Kiyotake, Miyazaki 889-1692, Japan

**Keywords:** LUBAC, CCl_4_ and APAP-induced hepatitis, inflammation, fibrosis, hepatocyte apoptosis

## Abstract

Linear ubiquitin chain assembly complex (LUBAC), composed of SHARPIN (SHANK-associated RH domain-interacting protein), HOIL-1L (longer isoform of heme-oxidized iron-regulatory protein 2 ubiquitin ligase-1), and HOIP (HOIL-1L interacting protein), forms linear ubiquitin on nuclear factor-κB (NF-κB) essential modulator (NEMO) and induces NF-κB pathway activation. SHARPIN expression and LUBAC formation were significantly reduced in the livers of mice 24 h after the injection of either carbon tetrachloride (CCl_4_) or acetaminophen (APAP), both of which produced the fulminant hepatitis phenotype. To elucidate its pathological significance, hepatic SHARPIN expression was suppressed in mice by injecting shRNA adenovirus via the tail vein. Seven days after this transduction, without additional inflammatory stimuli, substantial inflammation and fibrosis with enhanced hepatocyte apoptosis occurred in the livers. A similar but more severe phenotype was observed with suppression of HOIP, which is responsible for the E3 ligase activity of LUBAC. Furthermore, in good agreement with these in vivo results, transduction of Hepa1-6 hepatoma cells with SHARPIN, HOIL-1L, or HOIP shRNA adenovirus induced apoptosis of these cells in response to tumor necrosis factor-α (TNFα) stimulation. Thus, LUBAC is essential for the survival of hepatocytes, and it is likely that reduction of LUBAC is a factor promoting hepatocyte death in addition to the direct effect of drug toxicity.

## 1. Introduction

Recently, a novel head-to-tail linear type of ubiquitination was shown to play an essential role in nuclear factor-κB (NF-κB) activation [1,2,3,4,5]. The linear ubiquitin assembly complex (LUBAC) conjugates the linear polyubiquitin chain on NF-κB essential modulator (NEMO) and thereby activates the canonical NF-κB pathway through IκB kinase (IKK) complex activation [2]. Currently, LUBAC, which has a molecular weight that is approximately 600 kDa, is assumed to be comprised of SHANK-associated RH domain interacting protein in postsynaptic density (SHARPIN), the longer isoform of heme-oxidized IRP2 ligase-1 (HOIL-1L) and HOIL-1L interacting protein (HOIP) [3,4,5]. Although both HOIL-1L and HOIP possess E3 ligase activity, it is HOIP that serves as an E3 ligase for linear polyubiquitination. SHARPIN and HOIL-1L bind to HOIP via their ubiquitin-like (UBL) domain and have thus far been regarded as being important for the stability of LUBAC [3]. Previous reports indicate that NF-κB transcriptional activation leads to increased releases of several inflammatory cytokines including tumor necrosis factor-α (TNF-α), interleukin-6 (IL-6), and IL-1β in Kupffer cells as observed in non-alcoholic steatohepatitis (NASH) livers [6,7,8,9]. On the other hand, NF-κB activity also reportedly promotes the survival of hepatocytes via the inductions of anti-apoptosis factors such as inhibitors of apoptosis protein (IAPs), including X-linked IAP [10,11]. Thus, NF-κB is involved in both inflammation and cell survival.

In this study, first, mice were treated with either carbon tetrachloride (CCl_4_) or acetaminophen (APAP), and SHARPIN expression was revealed to be significantly downregulated in the livers of these acute hepatic injury models. We previously demonstrated that LUBAC formation is severely impaired mainly via markedly reduced SHARPIN expression in the NASH livers of mice fed a methionine choline deficient (MCD) diet for eight weeks [12]. In contrast to the MCD diet- or high-fructose diet-induced NASH model [13,14], CCl_4_ or APAP-induced liver injury occurred within a surprisingly short period (i.e., just 24 h after treatment), which accompanied a marked reduction of LUBAC formation.

Thus, we subsequently attempted to investigate the pathological significance of reduced LUBAC expression in the liver as well as in Hepa1-6 hepatoma cells by infection with the corresponding short-hairpin RNA (shRNA) adenoviruses. Importantly, livers deficient in SHARPIN or HOIP exhibited marked death of hepatocytes, which may have resulted in severe inflammation and fibrosis. Herein, we present data suggesting that reduction of LUBAC is involved in the development of CCl_4_ and APAP-induced hepatocyte death.

## 2. Results

### 2.1. SHANK-Associated RH Domain-Interacting Protein (SHARPIN) Expression Was Downregulated in the Livers of Carbon Tetrachloride (CCl_4_) and Acetaminophen (APAP) Induced Acute Liver Injury Models, and in Hepa1-6 Cells Treated with APAP

**Figure 1:**
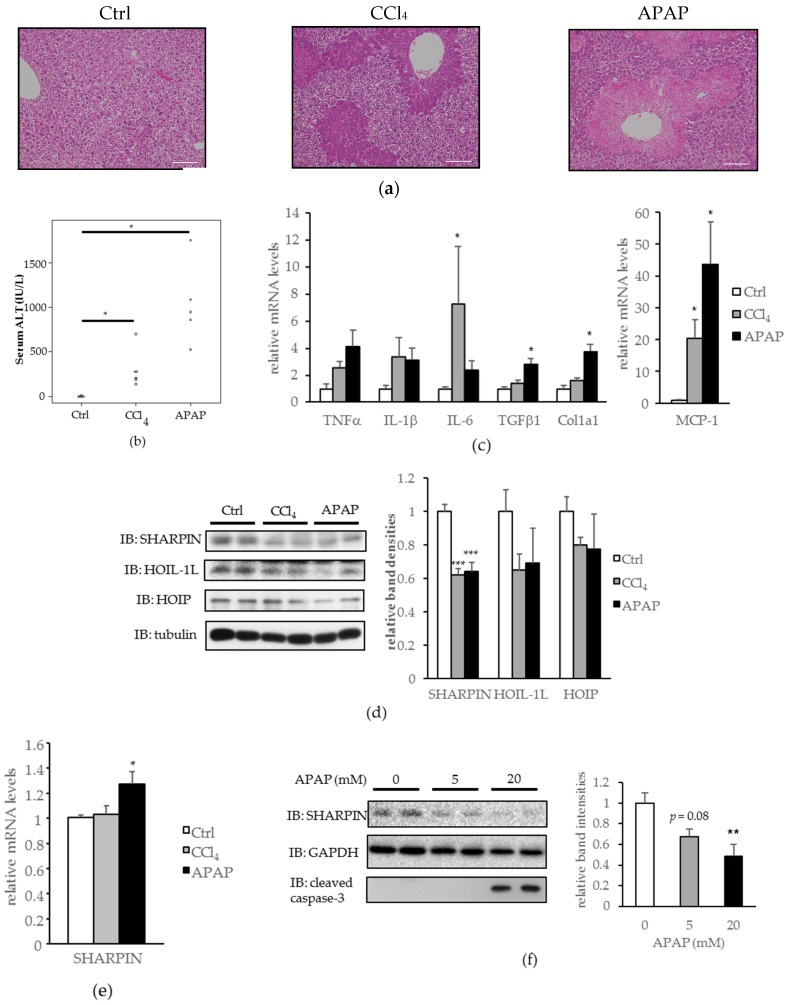
Downregulation of hepatic SHARPIN (SHANK-associated RH domain-interacting protein) by carbon tetrachloride (CCl_4_) and acetaminophen (APAP) induced acute liver injury in mice, as well as in Hepa1-6 cells treated with APAP. (**a**) Mice were injected with CCl_4_ (0.4 mL/kg) or APAP (300 mg/kg), and 24 h later their livers were removed and stained with hematoxylin and eosin (H-E). (Scale bar: 100 µm); (**b**) Serum alanine transaminase (ALT) level. (*n* = 5–6); (**c**) mRNA levels of inflammatory and pro-fibrogenic cytokines in the liver. (*n* = 5–6) (* *p* < 0.05); (**d**) Immunoblotting of each linear ubiquitin assembly complex (LUBAC) component in the liver. Relative band intensities after the adjustment for tubulin expression are shown as means + standard error (S.E.) (*n* = 5–6) (* *p* < 0.05, *** *p* < 0.001); (**e**) mRNA levels of SHARPIN. (*n* = 5–6) (* *p* < 0.05); (**f**) Immunoblotting of SHARPIN in Hepa1-6 cells after APAP stimulation for 24 h. Relative band intensities after the adjustment for glyceraldehyde-3-phosphate dehydrogenase (GAPDH) expression are shown as means + S.E. (*n* = 4) (** *p* < 0.01)

Mice were injected intraperitoneally with CCl_4_ or APAP according to the procedure described in the literature [15], and expression levels of each LUBAC component were then examined. The CCl_4_ and APAP injections both induced severe liver injuries, as shown by hematoxylin and eosin (H-E) staining (Figure 1a), serum alanine transaminase (ALT) elevation (Figure 1b), and increased mRNA levels of inflammatory and pro-fibrogenic cytokines (Figure 1c). Hepatic SHARPIN expression was significantly downregulated in both acute liver injury models (Figure 1d), without corresponding decreases in its mRNA levels (Figure 1e). HOIL-1L and HOIP amounts were also slightly decreased, although the differences did not reach statistical significance (Figure 1d).

Similar to the findings in the mouse livers, reduced SHARPIN expression was observed in Hepa1-6 cells (mouse hepatoma cells) treated with APAP for 24 h (Figure 1f). At the 20 mM concentration of APAP, reduction of SHARPIN was marked, and cleaved caspase-3, a marker of apoptosis, was detected.

These results suggest LUBAC deficiency as a possible underlying mechanism in the development of CCl_4_ and APAP-induced hepatocyte death.

### 2.2. Hepatic SHARPIN Knockdown Induces Apoptosis in Hepatocytes and Leads to Inflammation and Fibrosis in the Liver

The shRNA expressing adenoviruses targeting each LUBAC component were prepared to examine the influences of a LUBAC component being knocked down in the liver. As hepatic SHARPIN expression was significantly downregulated in the CCl_4_ and APAP induced acute liver injury models (Figure 1c), as well as in the MCD-diet fed NASH model [12], we first knocked down SHARPIN in the liver by injecting C57BL6/J mice with SHARPIN shRNA expressing adenovirus (Ad-SHARPIN shRNA) via the tail vein. LacZ shRNA expressing adenovirus (Ad-LacZ shRNA) was used as a control.

**Figure 2:**
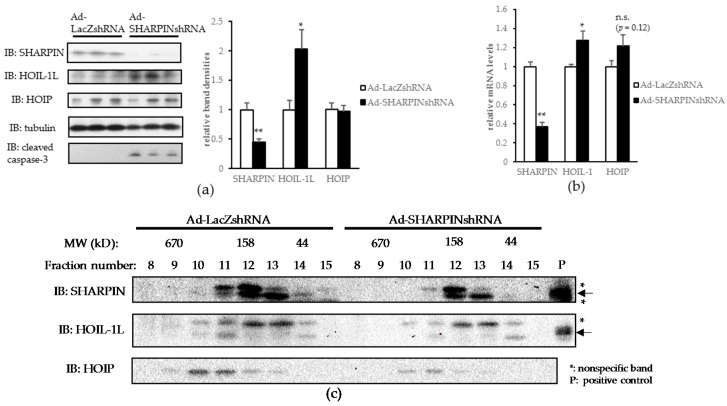

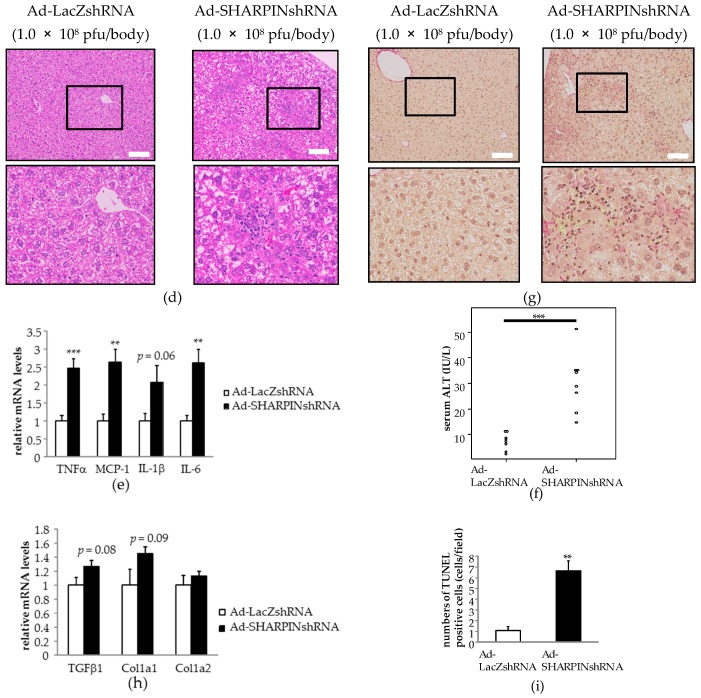
SHARPIN knockdown induces inflammation and fibrosis in the liver accompanied by increased apoptosis. (**a**) Immunoblotting of each LUBAC component in the liver. (*n* = 8) (* *p* < 0.05, ** *p* < 0.01); (**b**) mRNA levels of each LUBAC component in the liver. (*n* = 8) (* *p* < 0.05, ** *p* < 0.01); (**c**) Liver lysates were fractionated by gel filtration and then subjected to immunoblotting of each LUBAC component. Arrows: objective bands, asterisks: non-specific bands. P: positive controls (mouse total liver lysate for SHARPIN and lysate from Hepa1-6 cells transfected with mouse HOIL-1L plasmid for HOIL-1L); (**d**) H-E staining of the liver. Lower pictures are the enlarged versions of the framed portions. (Scale bar: 100 µm); (**e**) mRNA levels of inflammatory cytokines in the liver. (*n* = 7–8) (** *p* < 0.01, *** *p* < 0.001); (**f**) Serum ALT level. (*n* = 8) (*** *p* < 0.001); (**g**) picrosirius red staining of the liver. Lower pictures are the enlarged versions of the framed portions. (Scale bar: 100 µm); (**h**) mRNA levels of profibrogenic cytokines in the liver. (*n* = 7–8); (**i**) TUNEL (terminal deoxynucleotidyl transferase (TdT) dUTP nick-end labeling) staining of the liver was performed and TUNEL-positive cells were counted in three randomly selected fields (magnification field 200×) in each mouse. (** *p* < 0.01). PFU, plaque forming units. n.s., not significant.

In the livers of mice injected with Ad-SHARPIN shRNA (L-SHARPIN KD mice), both mRNA and protein levels of SHARPIN were significantly downregulated, whereas HOIL-1L expression was significantly upregulated probably as a compensatory response (Figure 2a,b). Gel filtration revealed all LUBAC components in the fraction corresponding to the LUBAC complex (~600 kD) to be decreased, suggesting LUBAC formation to have been destabilized in the livers of L-SHARPIN KD mice (Figure 2c).

In the livers of L-SHARPIN KD mice, immune cell infiltration was apparent (Figure 2d). Consistent with this finding, mRNA levels of inflammatory cytokines such as tumor necrosis factor-α (TNF-α), monocyte chemotactic protein-1 (MCP-1), and interleukin-6 (IL-6) were significantly higher than in control livers of L-SHARPIN KD mice (Figure 2e). Serum ALT was also mildly but significantly elevated in L-SHARPIN KD mice (Figure 2f). Picrosirius red staining revealed increased collagen deposition in the livers of L-SHARPIN KD mice (Figure 2g). Though not statistically significant, pro-fibrogenic cytokine expressions and collagen mRNAs also tended to rise (Figure 2h). Moreover, a significant increase in TUNEL (terminal deoxynucleotidyl transferase (TdT) dUTP nick-end labeling) positive cells (Figure 2i) suggested an increased susceptibility to apoptosis in the livers of L-SHARPIN KD mice, an observation also supported by an increased level of cleaved caspase-3 on immunoblotting (Figure 2a).

These results indicate that SHARPIN suppression in the liver can trigger inflammation, fibrosis, and apoptosis, all of which are characteristic features not only distinguishing NASH from simple steatosis [16], but also observed in acute liver injury models produced by administration of CCl_4_ or APAP (Figure 1c,f).

### 2.3. Hepatic HOIL-1L Interacting Protein (HOIP) Knockdown Also Induces Hepatocyte Apoptosis, Severe Inflammation, and Fibrosis in the Liver

**Figure 3:**
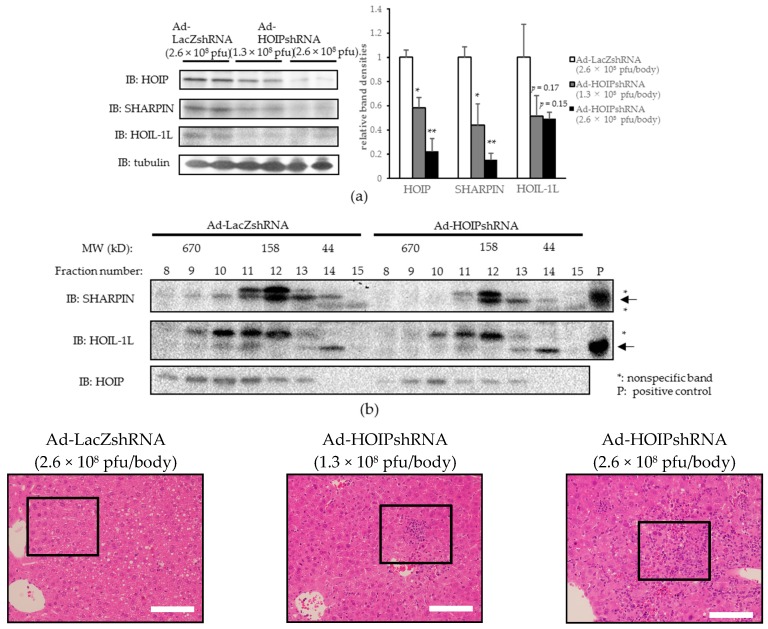

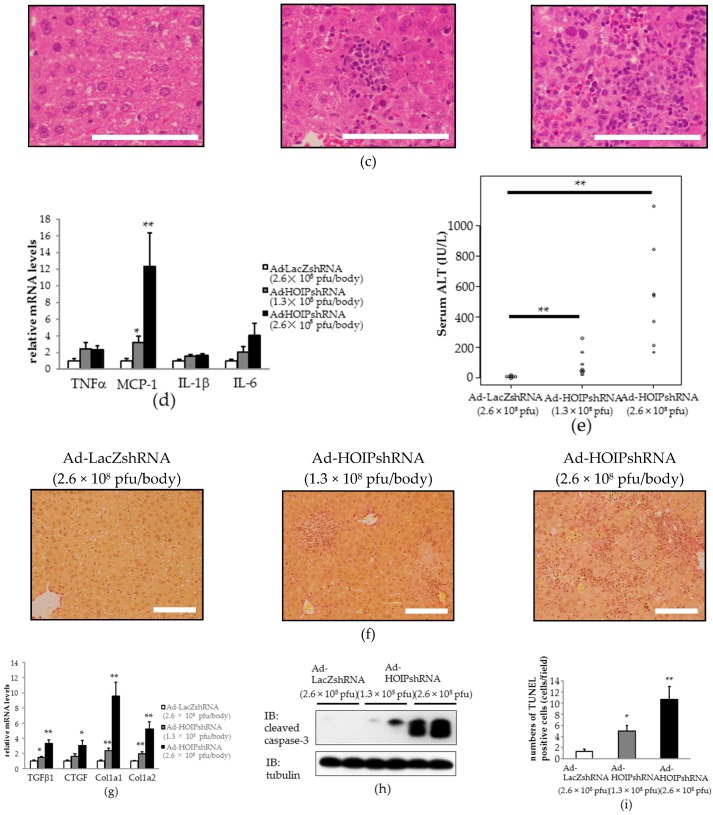
HOIL-1L interacting protein (HOIP) knockdown induces hepatocyte apoptosis, severe inflammation, and fibrosis, without steatosis in the liver. (**a**) Immunoblotting of each LUBAC component in the liver. (*n* = 4) (* *p* < 0.05, ** *p* < 0.01); (**b**) Liver lysates were fractionated by gel filtration and then subjected to immunoblotting of each LUBAC component. Arrows: objective bands, asterisks: non-specific bands. P: positive controls; (**c)** H-E staining of the liver. Lower pictures are the enlarged versions of the framed portions. (Scale bar: 100 µm); (**d**) mRNA levels of inflammatory cytokines in the liver. (*n* = 7–8) (* *p* < 0.05, ** *p* < 0.01); (**e**) Serum ALT level. (*n* = 7–8) (** *p* < 0.01); (**f**) picrosirius red staining of the liver. Lower pictures are the enlarged versions of the framed portions. (Scale bar: 100 µm); (**g**) mRNA levels of profibrogenic cytokines in the liver. (*n* = 7–8) (* *p* < 0.05, ** *p* < 0.01); (**h**) Immunoblotting of cleaved caspase-3 and tubulin, (**i**) TUNEL staining of the liver was performed and TUNEL-positive cells were counted in three randomly selected fields (magnification field 200×) in each mouse. (* *p* < 0.05, ** *p* < 0.01).

Among the components of LUBAC, it is HOIP that serves as an E3 ligase upon linear ubiquitination. To further elucidate the influence of hepatic LUBAC impairment in vivo, we next knocked down HOIP by injecting HOIP shRNA expressing adenovirus (Ad-HOIP shRNA) via the tail vein. In mice injected with Ad-HOIP shRNA (L-HOIP KD mice), the HOIP protein level was dose-dependently decreased in the liver by about 80% in those injected with the highest dose (2.6 × 10^8^ plaque forming units (pfu)/body) of Ad-HOIP shRNA (Figure 3a). Ad-HOIP shRNA injection did not affect HOIP expression levels in other tissues (Figure S1). The expressions of SHARPIN and HOIL-1L were also decreased in the livers of L-HOIP KD mice (Figure 3a), probably due to destabilization of LUBAC as a complex, an observation consistent with those of previous reports [17,18]. Destabilization of the LUBAC complex in the livers of L-HOIP KD mice was also confirmed by gel filtration, based on decreased amounts of each LUBAC component in the ~600 kD fraction (Figure 3b).

H-E staining revealed significantly and dose-dependently increased immune cell infiltration in the livers of L-HOIP KD mice as compared to control mice (Figure 3c). Monocyte chemotactic protein-1 (MCP-1) expression was significantly increased in the livers of L-HOIP KD mice, and though not statistically significant, mRNA levels of tumor necrosis factor-α (TNF-α), interleukin-1β (IL-1β), and interleukin-6 (IL-6) also tended to be increased in the livers of L-HOIP KD mice (Figure 3d). The serum ALT level was also significantly, and more markedly than in L-SHARPIN KD mice, increased in L-HOIP KD mice, reflecting severe hepatic inflammation (Figure 3e). Picrosirius red staining showed increased collagen deposition in the livers of L-HOIP KD mice, especially near the infiltrating immune cells (Figure 3f). The mRNA levels of tissue growth factor β1 (TGFβ1) and connective tissue growth factor (CTGF), which are essential pro-fibrogenic cytokines, were significantly increased in the livers of L-HOIP KD mice (Figure 3g). Collagen 1a1 and 1a2 expressions were also significantly increased (Figure 3g), results consistent with those of picrosirius red staining. Triglyceride contents were not increased in the livers of L-HOIP KD mice, which excludes the possibility of steatosis as a cause of inflammation in this model (data not shown).

Much as in L-SHARPIN KD mice, HOIP knockdown in the liver resulted in significant and dose-dependent increases in caspase-3 cleavage as compared to the controls (Figure 3h). TUNEL staining also revealed an apparent increase in apoptotic cells in the livers of L-HOIP KD mice (Figure 3i).

Taken together, our observations indicate that HOIP knockdown in the liver induces hepatocyte apoptosis and leads to severe inflammation and fibrosis, which is similar to, but presumably more severe, than that produced by SHARPIN knockdown in the liver.

### 2.4. Knockdown of Each Component of Linear Ubiquitin Chain Assembly Complex (LUBAC) Leads to Increased Tumor Necrosis Factor-α (TNFα)-induced Apoptosis in Hepatoma Cells In Vitro

**Figure 4:**
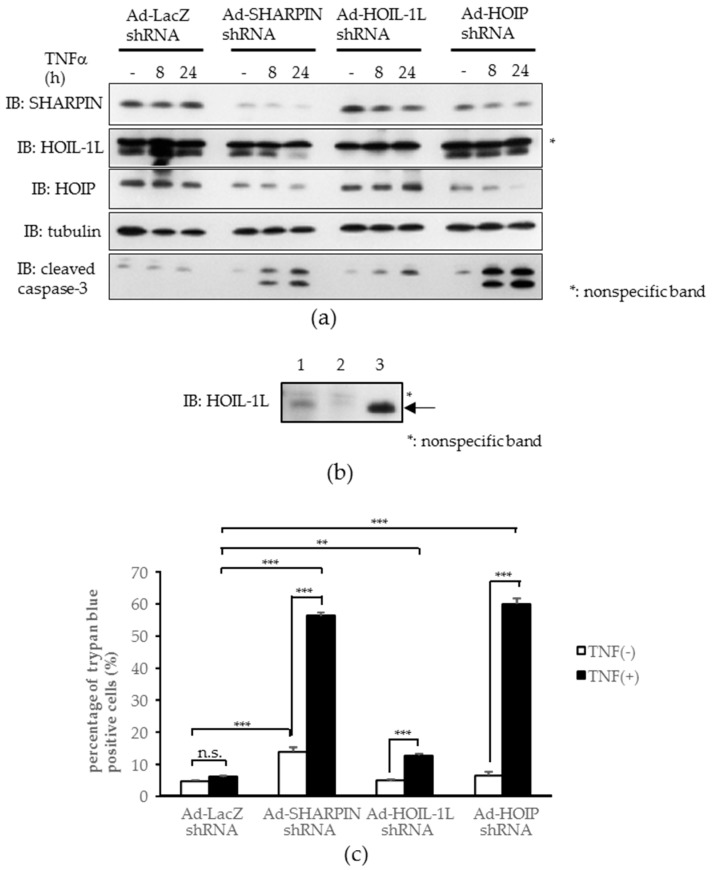
LUBAC insufficiency increases to tumor necrosis factor-α (TNFα)-induced apoptosis in Hepa1-6 cells. (**a**) Immunoblotting of each LUBAC component and cleaved caspase-3. Asterisks: non-specific bands; (**b**) Immunoblotting of HOIL-1L in Hepa1-6 cells. Lysates from Hepa1-6 cells, transduced with Ad-LacZ (1), Ad-HOIL-1L shRNA (2), or mouse HOIL-1L plasmid (3) were subjected to immunoblotting of HOIL-1L. Arrows: objective bands, asterisks: non-specific bands; (**c**) Quantitative cell death assay by trypan blue staining. (*n* = 4) (** *p* < 0.01, *** *p* < 0.001)

Since hepatocellular death can reportedly trigger various liver diseases [16,19] and the NF-κB pathway is well known to exert an inhibitory effect on apoptosis, we investigated whether LUBAC impairment renders hepatocytes susceptible to apoptosis in vitro. To examine whether apoptosis occurs in cultured cells, adenovirus-mediated knockdown of each LUBAC component was performed in Hepa1-6 cells. After TNFα treatment, cleavage of caspase-3 was increased in the cells infected with SHARPIN, HOIL-1L, or HOIP shRNA adenovirus, as compared with control LacZ shRNA adenovirus (Figure 4a) (the upper band of HOIL-1L was proven to be nonspecific, as shown in Figure 4b). Consistently, the quantitative cell death assay employing trypan blue staining revealed significantly increased cell death after TNFα treatment in cells infected with any one of the three LUBAC component shRNA adenoviruses, being especially marked in cells infected with SHARPIN or HOIP shRNA adenovirus (Figure 4c). These results indicate LUBAC-deficient hepatocytes to be highly susceptible to apoptosis, which supports our in vivo findings.

## 3. Discussion

To our knowledge, this is the first study to demonstrate the expressions of individual LUBAC components, especially SHARPIN, to be significantly decreased in mouse livers in response to treatment with CCl_4_ or APAP, agents widely used to create liver injury models. In contrast to the NASH model produced by eight weeks of MCD diet feeding [12], it should be noted that CCl_4_ or APAP-induced reduction of LUBAC formation occurred within just 24 h after treatment with these agents. Given that there were no significant decreases in their component mRNA levels, we can reasonably speculate that enhanced degradation would be the most likely mechanism underlying the observed reductions, though further study is necessary to clarify this issue.

APAP overdosage results in the formation of *N*-acetyl-*p*-benzoquinone imine (NAPQI), which covalently binds to glutathione (GSH) and depletes hepatic anti-oxidative capacity. Uncontrolled oxidative stress leads to hepatocyte cell death and the appearance of damage associated molecular patterns, a process which in turn activates Kupffer cells and results in the release of inflammatory cytokines and subsequent infiltration of neutrophils and macrophages [20,21]. Hepatotoxicity caused by CCl_4_ is also mediated via oxidative stress exerted by its degraded metabolites, trichloromethyl (CCl_3_) and trichloromethyl peroxyl (CCl_3_O_2_), both of which are unstable radicals [21].

We speculated that not only the toxicities of these agents themselves but also impaired LUBAC formation may contribute to the aggravation of liver injury. In fact, it was clearly shown that hepatic knockdown of either SHARPIN or HOIP by adenoviral transfer of the corresponding shRNA into mice produced severe inflammation and fibrosis accompanied by hepatocyte death, features typically observed in both drug-induced hepatitis and NASH livers. Similar results were obtained using the Hepa1-6 cell line. Knockdown of any one of the LUBAC components in Hepa1-6 cells induced apoptosis in the presence of TNFα-stimulation (Figure 4a). One difference from the data obtained with mouse livers was that no increase in caspase cleavage was apparent at baseline (i.e., without TNFα-stimulation) in Hepa1-6 cells (Figure 4a). This finding is in good agreement with those of a previous study showing that HOIP deficient cells tend to form complex-II, a cell death complex which involves RIP1, RIP3, FADD, cFLIP, and caspase-8, rendering HOIP deficient cells susceptible to cell death in response to TNFα stimulation [18].

In contrast, either SHARPIN or HOIP knockdown by adenoviral transfer of the corresponding shRNA into mouse livers was sufficient for rapid induction of massive hepatocyte death together with inflammation and fibrosis, with no additional stimulation being necessary. Gerlach et al. [5] reported the presence of nodular lymphocyte aggregates in the livers of *cpdm* mice (mice with a spontaneous *Sharpin* gene mutation), but these aggregates were absent when the *Tnf* gene was further knocked out in *cpdm* mice. In the mouse system, gut-derived bacterial endotoxins, such as lipopolysaccharide, have been regarded as an endogenous inducer of hepatic inflammation which exerts its effects by stimulating TNFα release from Kupffer cells, which is assumed to be a factor contributing to the progression from simple steatosis to NASH [22,23,24]. In addition to such endogenous inflammatory stimuli, it is possible that adenovirus infection, as used in our experiments, may contribute to apoptosis by functioning as a trigger, when HOIP or SHARPIN expression is suppressed. Thus, it is reasonable to speculate that LUBAC deficiency would lower the threshold of apoptosis and render hepatocytes susceptible to apoptosis, even when exposed to minor inflammatory stimuli such as gut-derived lipopolysaccharide, which in turn leads to inflammatory cell recruitment and hepatic stellate cell activation, thereby inducing inflammation and fibrosis in the liver [16]. It is noteworthy that the expression of IL-6, which is known to play a protective role against CCl_4_-induced hepatocyte apoptosis and fibrosis [25] and is reportedly important in the regenerative response to partial hepatectomy [26,27], was upregulated in the livers of both L-SHARPIN KD and L-HOIP KD mice concomitantly with other inflammatory cytokines (Figure 2e and Figure 3d), an observation ruling out the possibility that the enhancements of hepatic apoptosis and fibrosis in these mice were mediated via impaired IL-6 expression.

Two hepatic LUBAC-deficient murine models, L-SHARPIN KD mice and L-HOIP KD mice, were investigated to elucidate the role of LUBAC in the liver. Liver inflammation and fibrosis accompanied by hepatocyte apoptosis were observed in both of these KD murine models. l-SHARPIN KD mice showed a milder phenotype than l-HOIP KD mice, however, judging from serum ALT (Figure 2f and Figure 3e) and liver histology. Since the suppressive effects of our shRNA adenoviruses for HOIP and SHARPIN were both partial, these phenotypic differences are difficult to explain. Nevertheless, intriguingly, HOIL-1L expression was significantly upregulated on both the mRNA and the protein level in the livers of L-SHARPIN KD mice (Figure 2a,b). Thus, one possibility is that this upregulated HOIL-1L prevented apoptotic cell death to some degree by increasing residual LUBAC activity, but further study is necessary to clarify this issue.

Recent studies suggest circulating microRNAs, including miR-122, as potential biomarkers of drug-induced acute liver injuries [28,29]. Alteration of protein expression patterns in the liver in response to APAP administration has been also investigated using a proteomic approach [30,31]. It is intriguing whether these changes in microRNAs and protein expression patterns are observed in L-SHARPIN KD and L-HOIP KD mice as well, which also awaits further investigation.

In conclusion, to our knowledge, this is the first report showing that LUBAC formation, particularly the SHARPIN expression level, is reduced in the livers of mice with toxic agent-induced hepatitis. In addition, our findings strongly suggest impairments and/or reductions of LUBAC component functions to be a factor exacerbating hepatocyte death, in addition to the direct toxicity of the agent itself.

## 4. Materials and Methods

### 4.1. Animals and Experimental Protocols

C57BL6/J male mice, at seven to eight weeks of age, were fed normal chow diet. To prepare the mouse model with toxic agent-induced fulminant hepatitis, mice were intraperitoneally injected with 5 mL/kg CCl_4_ (8% in corn oil) or 300 mg/kg APAP (dissolved in warm saline) after being fasted for 20 h and were then sacrificed 24 h after the injection [15]. To reduce the hepatic expression levels of SHARPIN or HOIP, mice were injected via the tail vein with recombinant adenovirus encoding short-hairpin (sh) RNA for SHARPIN, HOIP, or LacZ as a control, and were sacrificed seven days after the injection. Liver-specific knockdown was confirmed by subjecting tissue lysates to Western blotting (Figure S1). HOIL-1L shRNA adenovirus was not used for the mouse experiments, since this virus suppressed the expression of HOIL-1L in vitro but not in mouse livers. The animals were handled in accordance with the Guidelines for the Care and Use of Experimental Animals published by Hiroshima University (Hiroshima, Japan), and the experimental protocols were approved by the Institutional Review Board of Hiroshima University (identification code: A16-35).

### 4.2. Histological Studies

Livers were fixed with 10% formaldehyde and embedded in paraffin. Sections were cut and stained with hematoxylin and eosin or picrosirius red. For TUNEL staining, either the Apoptag Peroxidase In Situ Apoptosis Detection Kit (Merck Millipore, Darmstadt, Germany) or the DeadEnd Colorimetric Apoptosis Detection System (Promega Corporation, Madison, WI, USA) was used according to the manufacturers’ instructions.

### 4.3. Serum Investigations

Whole blood samples were obtained from the heart and serum was collected after centrifugation at 1000× *g* for 30 min. Alanine aminotransferase (ALT) was assayed with the Transaminase CII Test (Wako, Osaka, Japan).

### 4.4. Cell Culture and Cell Death Assay

Hepa1-6 cells were maintained in Dulbecco’s modified Eagle’s medium (DMEM) containing 10% fetal bovine serum at 37 °C in 5% CO_2_ in air. At sub-confluence, Hepa1-6 cells were infected with LacZ, SHARPIN, HOIL-1L, or HOIP shRNA expressing adenoviruses and stimulated with 10 ng/mL TNFα for 8 or 24 h. Three days after the transduction, total lysates were prepared and then subjected to immunoblotting. Quantitative cell death assays were performed by staining dead cells with trypan blue. Two days after adenoviral infection, Hepa1-6 cells were stimulated with 10 ng/mL TNFα for 24 h. Cells were trypsinized, resuspended in culture medium, and stained by adding a 1/10 volume of 0.5% trypan blue. In the experiment designed to investigate the effect of APAP in vitro, Hepa1-6 cells were stimulated with 5 or 20 mM APAP for 24 h and total lysates were subjected to immunoblotting.

### 4.5. Antibodies

Antibodies were purchased from Cell Signaling Technology (cleaved caspase-3, GAPDH) and Santa Cruz (actin, α-tubulin). The antibodies against SHARPIN, HOIL-1L, and HOIP were prepared as previously described [12].

### 4.6. Western Blotting

Livers were homogenized in lysis buffer containing 50 mM Tris-HCl (pH 7.4), 150 mM NaCl, 1 mM etylenediaminetetraacetic acid (EDTA), 1% Triton X-100, 1 mM NaF, 1 mM Na_3_VO_4_, and 1 mM phenylmethylsulfonyl fluoride (PMSF). The lysates were incubated on ice for 30 min and then centrifuged at 15,000 rpm for 10 min and subsequently for 30 min at 4 °C. After adjusting the protein concentrations, the supernatants were mixed and boiled with sample buffer. Samples were electrophoresed with SDS (sodium dodecyl sulfate) -polyacrylamide gel, transferred to polyvinylidene difluoride (PVDF) membranes, and subjected to immunoblotting using Supersignal West Pico Substrate (Thermo Scientific, Waltham, MA, USA) or ImmunoStar LD (Wako).

### 4.7. RNAi Interference

For the individual knockdowns of mouse SHARPIN, HOIL-1L, and HOIP, we generated each shRNA expressing recombinant adenoviruses using the BLOCK-iT^TM^ U6 RNAi Entry Vector Kit and the BLOCK-iT^TM^ Adenoviral RNAi Expression System (Invitrogen). The top and bottom strands used to generate each of the shRNA expressing recombinant adenoviruses are as follows: SHARPIN shRNA, top strand 5′-CACCGCGGAAGCTGCAATTGATAGCCGAAGCTATCAATTGCAGCTTCCGC-3′, bottom strand 5′-AAAAGCGGAAGCTGCAATTGATAGCTTCGGCTATCAATTGCAGCTTCCGC-3′; HOIL-1L shRNA, top strand 5′-CACCGCCTATCTCTACCTGCTGTCACGAATGACAGCAGGTAGAGATAGGC-3′, bottom strand 5′-AAAAGCCTATCTCTACCTGCTGTCATTCGTGACAGCAGGTAGAGATAGGC-3′; HOIP shRNA, top strand 5′-CACCGGTCTTCTCAGCTCTCCAATACGAATATTGGAGAGCTGAGAAGACC-3′, bottom strand 5′-AAAAGGTCTTCTCAGCTCTCCAATATTCGTATTGGAGAGCTGAGAAGACC-3′.

### 4.8. Real-Time PCR (Polymerase Chain Reaction)

Total RNA from the liver was isolated using Sepazol reagent (Nacalai Tesque, Kyoto, Japan) and first-strand cDNA was obtained using the Verso cDNA Synthesis Kit (Thermo Scientific), according to the manufacturers’ instructions. Real-time PCR was performed using the CFX96 real-time PCR system (Bio-Rad, Hercules, CA, USA) with SYBR Premix Ex Taq (Takara-Bio, Kusatsu, Japan). The primers used are shown in Table 1.

### 4.9. Gel Filtration

Gel filtration was performed to detect LUBAC components forming the LUBAC complex, which could be eluted in fractions of higher molecular weight and thereby distinguished from those existing as monomers. Livers were homogenized in lysis buffer containing 50 mM Tris-HCl (pH 7.5), 1 mM MgCl_2_, 1 mM PMSF, 1 mM dithiothreitol (DTT), and protease inhibitor cocktail. After adding an equal volume of lysis buffer containing 300 mM NaCl, lysates were centrifuged at 15,000 rpm for 30 min. The supernatants were subjected to gel filtration using Superdex 200 10/300 GL (GE Healthcare, Chicago, IL, USA) and fractionated at 1 mL/min using ÄKTA explorer 10S (GE Healthcare). Proteins were precipitated with trichloroacetate and acetone from the collected samples, mixed and boiled with sample buffer, and subjected to western blotting.

### 4.10. Statistical Analysis

Statistical analyses were performed using EZR (Saitama Medical Center, Jichi Medical University, Saitama, Japan). Values are presented as means ± S.E. We used student’s unpaired *t*-test when comparing two groups, and one-way ANOVA or the Kruskal-Wallis test for multiple comparisons. We considered *p* < 0.05 to indicate a statistically significant difference.

## 5. Conclusions

LUBAC formation, especially the SHARPIN expression level, was reduced in the livers of mice with CCl_4_- or APAP-induced hepatitis. Furthermore, either SHARPIN or HOIP knockdown by adenoviral transfer of the corresponding shRNA into mouse livers rapidly induced massive hepatocyte death together with inflammation and fibrosis. Therefore, the LUBAC impairment observed in the livers of CCl_4_- or APAP-induced hepatitis may be a factor exacerbating hepatocyte death, in addition to the direct toxicity of the agent itself.

## Figures and Tables

**Table 1:** Primer sets for real-time PCR.

Primer Sets for Real-Time PCR
mSHARPIN-F: CCTGTGTATGCCTGAACGAA
mSHARPIN-R: AGAGGATCCCAAGCACAGG
mHOIL-1-F: TCTCCCCAACACAGGACATC
mHOIL-1-R: AAATGGTGACGGTGTGCAT
mHOIP-F: CCCAGTGTCACCAGACCTTC
mHOIP-R: CCTCACAACTCCGTCCTCTG
mTNFα-F: GAACTGGCAGAAGAGGCACT
mTNFα-R: AGGGTCTGGGCCATAGAACT
mMCP-1-F: AGGTCCCTGTCATGCTTCTG
mMCP-1-R: TCTGGACCCATTCCTTCTTG
mIL-1β-F: TGACGGACCCCAAAAGATG
mIL-1β-R: TGGACAGCCCAGGTCAAAG
mIL-6-F: TCGTGGAAATGAGAAAAGAGTTG
mIL-6-R: AGTGCATCATCGTTGTTCATACA
mTGF-β1-F: AAGTTGGCATGGTAGCCCTT
mTGF-β1-R: GCCCTGGATACCAACTATTGC
mCTGF-F: AGGGCCTCTTCTGCGATTTC
mCTGF-R: CATTTCCCAGGCAGCTTGAC
mCollagen1a1-F: TCATCGTGGCTTCTCTGGT
mCollagen1a1-R: GACCGTTGAGTCCGTCTTTG
mCollagen1a2-F: CCGTGCTTCTCAGAACATCA
mCollagen1a2-R: GAGCAGCCATCGACTAGGAC

## Supplementary Materials

Supplementary materials can be found at www.mdpi.com/1422-0067/18/2/326/s1.

## Abbreviations

LUBAC	Linear ubiquitin chain assembly complex
SHARPIN	SHANK-associated RH domain-interacting protein
HOIL-1L	Longer isoform of heme-oxidized iron-regulatory protein 2 ubiquitin ligase-1
HOIP	HOIL-1L interacting protein
NASH	Non-alcoholic steatohepatitis
APAP	Acetaminophen
