# Supplementary material for: Reduced SHARPIN and LUBAC Formation May Contribute to CCl4- or Acetaminophen-Induced Liver Cirrhosis in Mice
Source: Int J Mol Sci. 2017 Feb 4;18(2):326. doi: 10.3390/ijms18020326 (PMC5343862; doi:10.3390/ijms18020326)
Supplement: Supplementary file 1 [file ijms-18-00326-s001.pdf]

Supplementary Materials: Reduced SHARPIN and LUBAC Formation May Contribute to CCl_4- or Acetaminophen-Induced Liver Cirrhosis in Mice

Takeshi Yamamotoya, Yusuke Nakatsu, Yasuka Matsunaga, Toshiaki Fukushima, Hiroki Yamazaki, Sunao Kaneko, Midori Fujishiro, Takako Kikuchi, Akifumi Kushiyama, Fuminori Tokunaga, Tomoichiro Asano and Hideyuki Sakoda

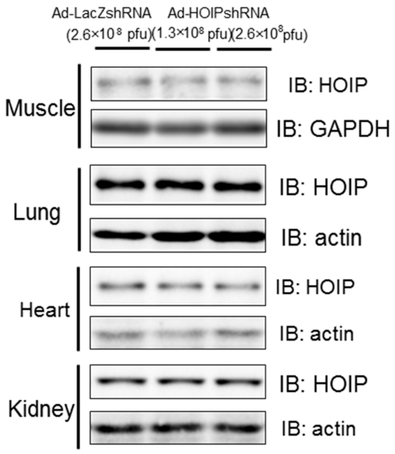

Figure S1. HOIP expression level was unchanged in extrahepatic tissues of L-HOIP KD mice.
